# Gut microbiota linked to hydrocephalus through inflammatory factors: a Mendelian randomization study

**DOI:** 10.3389/fimmu.2024.1372051

**Published:** 2024-07-15

**Authors:** Yingjie Shen, Changyu Li, Xi Zhang, Yaolou Wang, Haopeng Zhang, Zhao Yu, Binbin Gui, Renjie Hu, Qi Li, Aili Gao, Hongsheng Liang

**Affiliations:** ^1^ Department of Neurosurgery, National Health Commission Key Laboratory of Cell Transplantation, The First Affiliated Hospital of Harbin Medical University, Harbin, Heilongjiang, China; ^2^ Department of Neurosurgery, Hainan Cancer Hospital, Haikou, Hainan, China; ^3^ School of Life Science, Northeast Agricultural University, Harbin, Heilongjiang, China

**Keywords:** gut microbiota, hydrocephalus, inflammation, mediator, Mendelian randomization

## Abstract

**Background:**

The gut microbiota (GM) has been implicated in neurological disorders, but the relationship with hydrocephalus, especially the underlying mechanistic pathways, is unclear. Using Mendelian randomization (MR), we aim to discover the mediating role of inflammatory factors in the relationship between GM and hydrocephalus.

**Methods:**

After removing confounders, univariable and multivariable MR analyses were performed using summary statistics to assess the causal relationships between GM, inflammatory factors (IL-17A and IL-27), and types of hydrocephalus. Meta-analyses were used to reconcile the differences in MR results between different hydrocephalus sources. Finally, mediator MR analyses were applied to determine the mediating effect of inflammatory factors. Various sensitivity analysis methods were employed to ensure the reliability and stability of the results.

**Results:**

After correction for *P*-values, *Firmicutes* (*phylum*) (OR, 0.34; 95%CI, 0.17–0.69; *P* = 2.71E-03, *P*_FDR_ = 2.44E-02) significantly reduced the risk of obstructive hydrocephalus. The remaining 18 different taxa of GM had potential causal relationships for different types of hydrocephalus. In addition, *Firmicutes* (*phylum*) decreased the risk of obstructive hydrocephalus by increasing levels of IL-17A (mediating effect = 21.01%), while *Eubacterium ruminantium group* (*genus*) increased the risk of normal-pressure hydrocephalus by decreasing levels of IL-27 (mediating effect = 7.48%).

**Conclusion:**

We reveal the connection between GM, inflammatory factors (IL-17A and IL-27), and hydrocephalus, which lays the foundation for unraveling the mechanism between GM and hydrocephalus.

## 1 Introduction

Hydrocephalus is an old category of disorder that has been documented since ancient times. It is characterized by enlargement of the ventricular system due to an imbalance between the production and absorption of cerebrospinal fluid (CSF) (1). The healthcare burden of hydrocephalus is enormous in both developing and developed countries. Although surgery can reduce CSF production or facilitate shunting, the failure rate of surgery and the long-term infection rate cannot be ignored (2). The processes leading to impaired circulation of CSF remain unknown. However, microscopic explorations beyond the primary lesion may offer insights into the development and aggravation of hydrocephalus. According to recent studies, choroidal arteries trigger cytokine storms that promote hydrocephalic developments in cases of posthemorrhagic hydrocephalus. Neuroinflammation is regarded as a crucial factor in the development of hydrocephalus (3).

The gut microbiota (GM) relies on the intestinal environment to carry out immune, metabolic, and endocrine functions. Emerging studies have indicated the GM’s crucial role in controlling T cell homeostasis and neuroinflammation in neurological disorders (4). The gut-brain axis linking the gut and nervous system is becoming increasingly acknowledged. Previous research demonstrates that the GM has a role in various neurological conditions, such as Alzheimer’s disease, ischemic stroke, epilepsy, and Parkinson’s disease (5, 6). Furthermore, a correlation between GM and neuroinflammation following a stroke has been established. It is not just altering the GM composition after stroke; transferring specific gut microbes into animal models after hemorrhage has also reduced neuroinflammation (4). Thus, a correlation between the gut and neurological inflammation-related disorders may exist.

Interleukins (IL) is a large family with protective immune and pro-inflammatory mediators among the many inflammatory factors. It has now been found that some of the interleukins may have bidirectional physiopathologic roles, such as IL-17A and IL-27. IL-17A was the first member of the IL-17 family to be identified. In addition to its ability to induce cellular activation to produce inflammatory mediators, IL-17A has been found to play a central role in protective immunity against Candida and other fungal pathogens (7). In addition, IL-17A drives epithelial hypoxia-Inducible factor 1-α to promote wound repair via glycolysis (8). IL-27, on the other hand, has been found to activate multiple signaling cascades that promote/inhibit different TH cell activation and thus produce opposite immune effects, respectively (9). Also, IL-27 is associated with neurological pathologies, including myasthenia gravis, Guillain-Barre syndrome, and ischemia-reperfusion, and may even play a neuroprotective role in reducing neuroinflammation (10–12).

The secretions of GM found so far can interfere with the expression of inflammatory factors and thus contact distant tissues (13). Mendelian randomization (MR) is a new way of exploring causality. Since any given allele has the same chance of being randomly inherited in a person, the causality of MR studies is plausible (14). However, we need to be aware of studies elaborating on the relationship between gut microbiome, inflammatory factors, and hydrocephalus. Therefore, we will perform multiple MR analyses with the help of Genome-Wide Association Studies (GWAS) to further analyze the causal relationship between GM, inflammatory factors, and hydrocephalus.

## 2 Materials and methods

### 2.1 Study design and ethics statement

**Figure 1:**
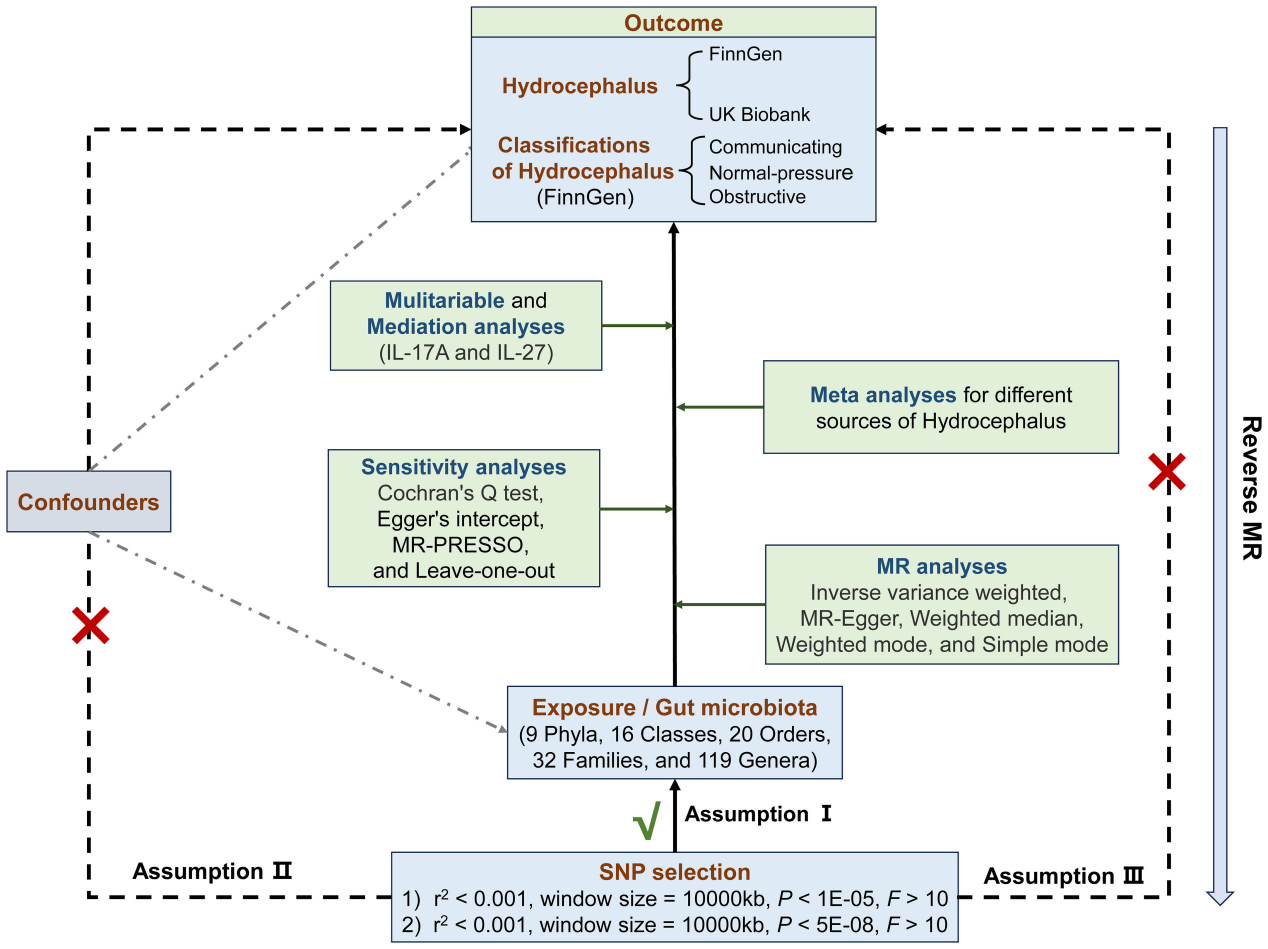
Flowchart of the Mendelian randomization and three key assumptions. MR, Mendelian randomization; SNP, single nucleotide polymorphism; MR-PRESSO, MR pleiotropy residual sum and outlier.

In all analyses, we used single nucleotide polymorphisms (SNPs) as instrumental variables (IVs) and relied on three main assumptions: (1) IVs are strongly associated with exposure; (2) IVs are not associated with confounders; and (3) IVs can only influence outcome through exposure (15). The second and third assumptions, collectively referred to as independence of horizontal pleiotropy, were assessed using various statistical methods. Initially, we chose the SNPs based on a locus-wide significance threshold (*P*< 1 × 10^-5^) and conducted bidirectional MR analyses to investigate the association between GM and hydrocephalus. Secondly, we reselected SNPs based on a genome-wide statistical significance cutoff (*P*< 5 × 10^-8^) for GM to enhance the precision of the causal link between GM and hydrocephalus. Subsequently, we employed meta-analysis to coordinate differences in the results of MR analyses derived from hydrocephalus in different cohorts. Lastly, we conducted mediator MR analyses to elucidate whether GM impacts hydrocephalus through inflammatory factors (16) (**Figure 1**).

We did not require additional ethical approval since we used publicly available data, which had already received approval from the appropriate ethical and institutional review boards. This study is reported in accordance with the Strengthening the Reporting of Observational Studies in Epidemiology Using Mendelian Randomization guidelines (STROBE-MR) (17) (**Supplementary Table S1**).

### 2.2 Data source

We obtained the GWAS dataset of the human GM from the MiBioGen consortium. This multi-ethnic, genome-wide analysis focuses on human autosomal genetic variation and the GM to investigate host-genetics-microbiome associations (18). From this comprehensive study, we identified 211 bacterial taxa: 9 phyla, 16 classes, 20 orders, 35 families (3 unknown), and 131 genera (12 unknown), resulting in 196 bacteria representing gut species in our analyses (details in **Supplementary Table S2**).

We initially obtained hydrocephalus GWAS data based on ICD-10 diagnostic criteria (G91) from the FinnGen consortium. An additional data source on hydrocephalus came from a study of a generalized linear mixed-model association tool on samples from the UK Biobank. For detailed analysis, we acquired GWAS data from FinnGen for specific hydrocephalus types based on ICD-10 classifications: communicating hydrocephalus (G91.0), normal-pressure hydrocephalus (G91.2), and obstructive hydrocephalus (G91.1). IL-17A was derived from measurements of 2,994 plasma proteins from 3,301 individuals with a total SNP number of 10,534,735 (19), whereas IL-27 was derived from measurements of 90 proteins from 21,759 participants in 13 cohorts with a total SNP number of 13,102,608 (20). Notably, all populations in these datasets were of European descent (**Supplementary Table S2**).

### 2.3 Selection of SNPs

First, we used SNPs with *P*< 1 × 10^-5^ as the initial genetic IVs for all MR analyses, which was determined to be the optimal threshold for the MR studies associated with GM as well as the other exposures in this study to maximize the amount of genetic variation explained by genetic predictors and to increase the number of SNPs eligible for sensitivity analysis (21). Second, we selected IVs for GM with a genome-wide significance cutoff (*P*< 5 × 10^-8^) to understand the corresponding exposure (22, 23). Subsequently, we applied linkage disequilibrium (r^2^< 0.001, window size = 10,000 kb) to exclude non-compliant SNPs (24). Also, we used *F*-statistic values to represent the intensity of IVs:


$$F=\frac{R^{2}(n-1-k)}{(1-R^{2})k}$$

with *R* ^2^ indicating the degree of variance elucidated by the instruments, *n* signifying the sample size, and *k* representing the number of chosen IVs, with *F*-statistic values< 10 were excluded (25). We removed SNPs significantly associated with the outcome based on the criterion: *P*_outcome_< *P*_exposure_. Finally, we excluded SNPs with palindromic structures to ensure alignment of exposure and outcome alleles.

### 2.4 Statistical analysis

In the MR analyses, we employed five MR-related statistical methods: inverse variance weighted (IVW), MR-Egger regression, weighted median, weighted mode, and simple mode. The IVW method enables the estimation of the causal effect of exposure on the outcome by integrating ratio estimates for each SNP (26). It transforms MR estimates into a weighted regression of SNP-outcome effects against SNP-exposure effects. IVW provides the most precise estimates among the methods considered, assuming all SNPs are valid instruments. If the β values of other methods were consistent, a significant result (*P*< 0.05) was obtained by the IVW method, and there is no pleiotropy and heterogeneity can be considered positive, even if other methods did not yield significant results (27). MR-PRESSO was also used to augment the robustness of the results. If the causal estimate conforms with the beta value of the method above and the *P*-value is less than 0.05, it enhances the robustness of the results.

We applied a significance level of *P*< 0.05 to assess heterogeneity and horizontal pleiotropy. Initially, we used Cochran’s Q test to evaluate heterogeneity (24). Then, we conducted the MR Egger’s intercept test and the global test of MR-PRESSO to assess horizontal pleiotropy. MR-PRESSO was used to identify outliers in IVs. If any outliers were found, we re-ran the MR analysis after excluding them. Additionally, we performed leave-one-out analyses by systematically excluding each instrumental SNP and re-executing the IVW analyses (28). Finally, we would not consider the causal relationship valid if horizontal pleiotropy was present and we could not correct for outliers.

For each outcome, we corrected the *P*-values for the false discovery rate (FDR) based on different categories of GM and inflammatory factors, resulting in a corresponding *P*_FDR_. In our analysis, a significant causal relationship between exposure and outcome is considered when *P<* 0.05 and *P*_FDR_< 0.1, whereas a potential association is suggested when *P*< 0.05 and *P*_FDR_ ≥ 0.1 (29, 30).

When types of hydrocephalus were used as outcomes for exposures causally related to hydrocephalus, we searched the PhenoScanner database for common confounders associated with hydrocephalus to minimize their interference (including hypertension, BMI, and cholesterol) (31). After removing these associated SNPs, we repeated the MR and sensitivity analyses.

### 2.5 Replication and meta-analysis for hydrocephalus

When GM was used as the exposure and hydrocephalus (G91) as the outcome, we used hydrocephalus data from the UK Biobank as additional replication analyses. We conducted meta-analyses to consolidate the results with causality from FinnGen and UK Biobank sets. The heterogeneity of meta-analyses was checked using I^2^ and corresponding *P*-values.

### 2.6 Mediation analysis

**Figure 2:**
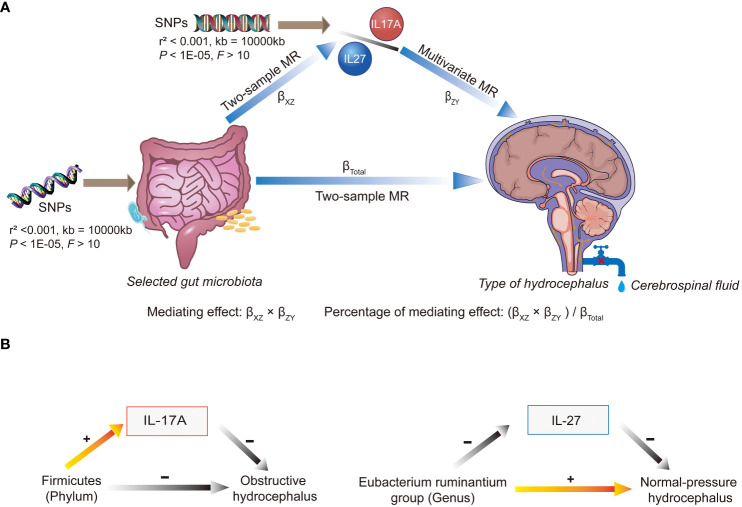
An overview chart of mediation analysis. SNP, single nucleotide polymorphism; MR, Mendelian randomization. **(A)** When multiple gut microbiota taxa were used as exposures, different microbiota affected the different types of hydrocephalus via IL-17A or IL-27. Mediating effects were calculated from beta values at multiple stages. **(B)** Elevated abundance of *Firmicutes* (*phylum*) reduced the risk of obstructive hydrocephalus by increasing levels of IL-17A. On the other hand, the *Eubacterium ruminantium group* (*genus*) increased the risk of normal-pressure hydrocephalus by decreasing levels of IL-27.

Regarding mediation, initially, we recorded the causality between GM and classifications of hydrocephalus as the total effects (β_Total_). Next, we examined the causal relationships between inflammatory factors (IL-17A, IL-27) and various classifications of hydrocephalus using two-sample MR analyses. Building on the previous information, the first step in the two-step MR was to assess the causal relationships of GM on inflammatory factors (IL-17A, IL-27), denoted by β_XZ_. The second step was to evaluate the causal relationships of inflammatory factors on classifications of hydrocephalus by multivariable MR (MVMR), denoted by β_ZY_. β_XZ_ × β_ZY_ denotes the mediating effect, and (β_XZ_ × β_ZY_)/β_Total_ denotes the percentage of this mediating effect. Mediating effects are considered to exist if (β_XZ_ × β_ZY_) and β_Total_ share consistent directions (as shown in **Figure 2A**).

All statistical analyses were conducted with R (version 4.3.0), and MR analyses were executed using the TwosampleMR, Mendelian Randomization, MVMR, and MR-PRESSO R packages.

## 3 Results

### 3.1 Causal relationship between gut microbiota and hydrocephalus (*P* < 1 × 10^-5^)

**Figure 3:**
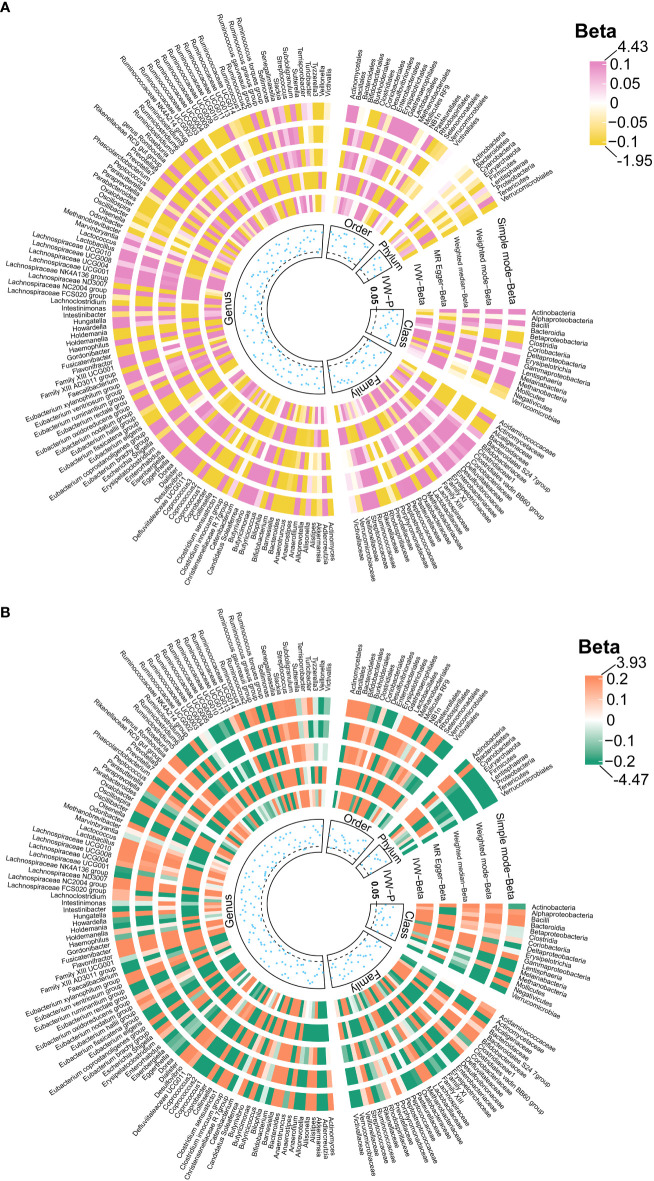
Causal effects of the gut microbiota on hydrocephalus **(A)** and communicating hydrocephalus **(B)** based on MR analyses. The data source for hydrocephalus derived from FinnGen. From outside to inside, the beta values of simple mode, weighted mode, weighted median, MR- Egger, inverse variance weighted, and *P* -value of inverse variance weighted are represented, respectively. MR, Mendelian randomization; IVW, Inverse variance weighted.

**Figure 4:**
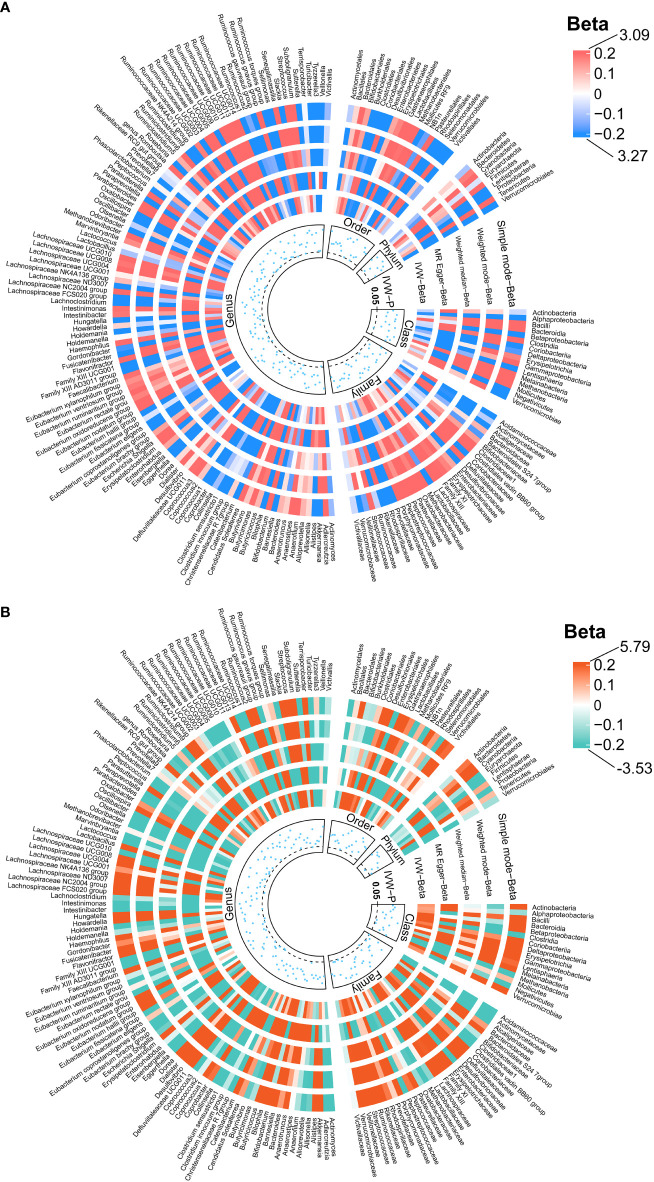
Causal effects of the gut microbiota on normal-pressure hydrocephalus **(A)** and obstructive hydrocephalus **(B)** based on MR analyses. The data source for hydrocephalus derived from FinnGen. From outside to inside, the beta values of simple mode, weighted mode, weighted median, MR- Egger, inverse variance weighted, and *P* -value of inverse variance weighted are represented, respectively. MR, Mendelian randomization; IVW, Inverse variance weighted.

**Figure 5:**
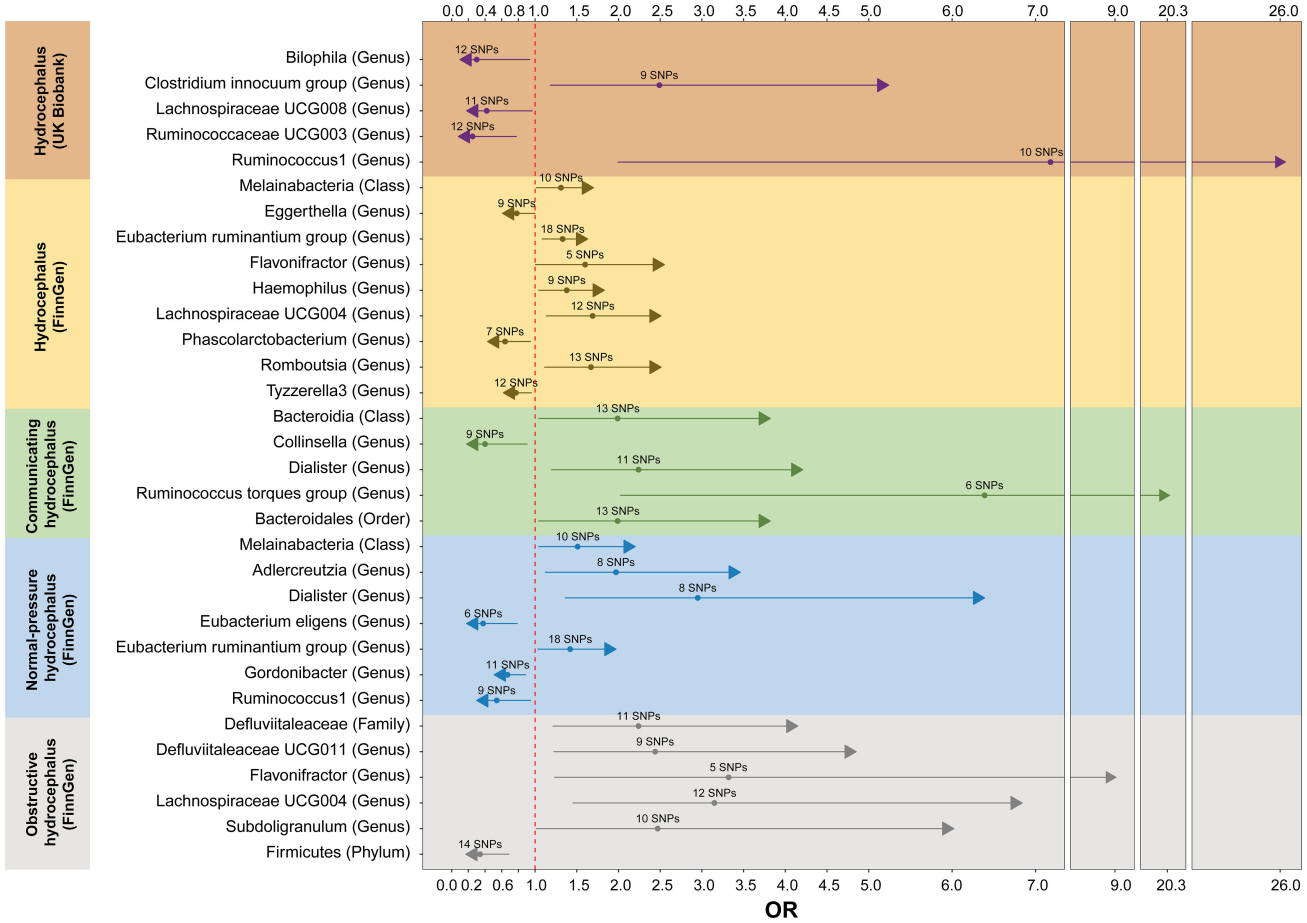
The forest plot of MR analyses between gut microbiota and hydrocephalus when selected instrumental variables with a locus-wide significance threshold (*P*< 1 × 10^-5^). Different color blocks represent different types of hydrocephalus as outcomes. The method used for MR analyses is inverse variance weighted. MR, Mendelian randomization; OR, Odds ratio.

We identified 192 distinct bacterial taxa for analyses following a rigorous IVs screening process. Then, we selected the corresponding SNPs for hydrocephalus (FinnGen) (n = 2029), communicating hydrocephalus (FinnGen) (n = 2042), normal-pressure hydrocephalus (FinnGen) (n = 2028), and obstructive hydrocephalus (FinnGen) (n = 2032), and hydrocephalus (UK Biobank) (n =2064). The *F*-values of all IVs were greater than 10, eliminating the bias of weak instruments. The results of MR analysis for GM and FinnGen-derived hydrocephalus are shown in **Figures 3** and **4**. After removing the confounders-related SNPs (**Supplementary Tables S3**, **S4**), we found that only *Firmicutes* (*phylum*) had a significant causal relationship with obstructive hydrocephalus (FinnGen) according to the criteria, indicating a protective effect (OR, 0.34; 95%CI, 0.17–0.69; *P* = 2.71E-03; *P*_FDR_ = 2.44E-02) (Shown in **Figure 5** and **Supplementary Table S5**).

For hydrocephalus (FinnGen), we identified several GMs with potential causal relationships. Specifically, *Melainabacteria* (*class*), *Eubacterium ruminantium group* (*genus*), *Flavonifractor* (*genus*), *Haemophilus (genus)*, *Lachnospiraceae UCG004* (*genus*), and *Romboutsia* (*genus*) potentially increased the risk of hydrocephalus. While *Eggerthella* (*genus*), *Phascolarctobacterium* (*genus*), and *Tyzzerella3* (*genus*) may be protective against it (**Figure 5** and **Supplementary Table S5**).

In assessing the causal effects of communicating hydrocephalus (FinnGen), several genetically predicted GMs were identified as potentially influential after removing the confounders-related SNPs (rs35866622 and rs2016057). *Collinsella* (*genus*) exhibited a potential protective effect against communicating hydrocephalus. In contrast, *Bacteroidia* (*genus*), *Dialister* (*genus*), *Ruminococcus torques group* (*genus*), and *Bacteroidales* (*order*) were associated with a potentially increased risk of communicating hydrocephalus (**Figure 5** and **Supplementary Table S5**).

For normal-pressure hydrocephalus (FinnGen), after removing the confounders-related SNP (rs10167839), we found that *Eubacterium eligens* (*genus*), *Gordonibacter* (*genus*), and *Ruminococcus1* (*genus*) exerted potentially protective effects. Conversely, *Melainabacteria* (*class*), *Adlercreutzia* (*genus*), *Dialister* (*genus*), and *Eubacterium ruminantium group* (*genus*) were associated with a potentially increased risk of normal-pressure hydrocephalus (**Figure 5** and **Supplementary Table S5**).

Regarding obstructive hydrocephalus (FinnGen), besides the significant protective effect of *Firmicutes (phylum)*, we found that *Defluviitaleaceae* (*family*), *Defluviitaleaceae UCG011* (*genus*), *Flavonifractor* (*genus*), *Lachnospiraceae UCG004* (*genus*), and *Subdoligranulum* (*genus*) potentially increased the risk of obstructive hydrocephalus (**Figure 5** and **Supplementary Table S5**).

Finally, to perform replication analyses, we used UK Biobank-derived data on hydrocephalus as the outcome. The results suggest that the *Clostridium innocuum group* (*genus*) and *Ruminococcus1* (*genus*) potentially increased the risk of hydrocephalus. In contrast, *Bilophila* (*genus*), *Lachnospiraceae UCG008* (*genus*), and *Ruminococcaceae UCG003* (*genus*) potentially decreased the risk of hydrocephalus (**Figure 5** and **Supplementary Table S5**).

In the sensitivity analyses, the *P*-values of Cochran’s Q tests were greater than 0.05, indicating no significant heterogeneity (**Supplementary Table S6**). In the MR-PRESSO test for *Dialister (genus)* and normal-pressure hydrocephalus (FinnGen), we identified two outlier SNPs. After removing these outliers and re-running all analyses, the MR-PRESSO results were satisfactory, further enhancing the robustness of the results (**Supplementary Table S7**). Additionally, the leave-one-out analyses showed that none of the identified causal associations were influenced by any single IV (**Supplementary Table S8**).

### 3.2 Causal relationship of hydrocephalus on selected gut microbiota

We performed reverse MR analyses using different types of hydrocephalus as exposures, selecting corresponding IVs to explore their causal effects on the various microbiotas mentioned.

The results of the selected SNPs and reverse MR analyses are provided in **Supplementary Tables S9** and **Supplementary Tables S10**. Based on the criteria, we found no significant results. That is, the abundance of selected GM did not change after hydrocephalus. In subsequent sensitivity analyses, despite no detected causality, we found heterogeneity in the causality assessment between the Eubacterium ruminantium group (genus) and hydrocephalus (FinnGen). Similar assessments were found for *Dialister* (*genus*) and normal-pressure hydrocephalus (FinnGen). Therefore, we used multiplicative random effects to represent the IVW results. We also identified a few outliers using MR-PRESSO and re-ran the MR analysis after excluding these abnormal SNPs. As shown in **Supplementary Tables S11** and **S12**, the results indicate no significant horizontal pleiotropy and no noteworthy effects on individual IV results.

### 3.3 Causal relationship between gut microbiota and hydrocephalus (*P* < 5 × 10^-8^)

**Figure 6:**
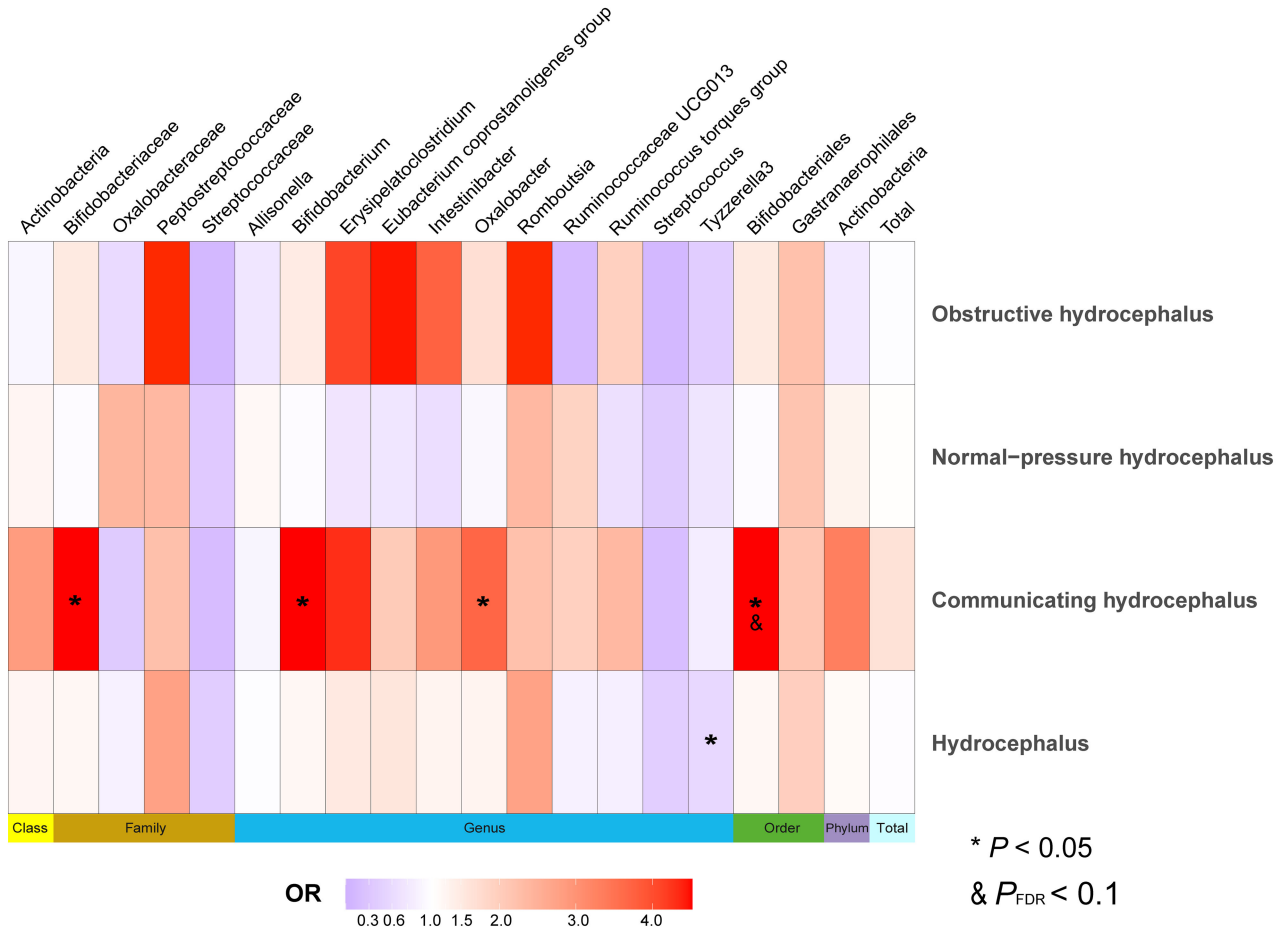
Heatmap of MR analysis between gut microbiota and hydrocephalus when selected instrumental variables with a genome-wide threshold of statistical significance (*P*< 5 × 10^-8^). Data on different types of hydrocephalus were obtained from FinnGen. Different color blocks represent different odds ratio values. The methods used for MR analyses include Wald ratio and Inverse variance weighted. “Total” indicates exposure of the entire gut microbiota. MR, Mendelian randomization; OR, Odds ratio.

We selected SNPs meeting genome-wide statistical significance criteria for individual and total GMs, as shown in **Supplementary Table S13**. After removing confounder-associated SNPs (**Supplementary Table S14**), we found that *Tyzzerella3* (*genus*) served as a potential protective factor against hydrocephalus (FinnGen). *Bifidobacteriales (order)* (OR, 8.56; 95%CI, 1.20–61.25; *P* = 2.75E-02; *P*_FDR_ = 5.50E-02) significantly increased the risk of communicating hydrocephalus (FinnGen), while *Bifidobacteriaceae* (*family*), *Bifidobacterium* (*genus*), and *Oxalobacter* (*genus*) were identified as potential risk factors for communicating hydrocephalus (FinnGen). Additionally, when hydrocephalus (UK Biobank) served as an outcome, *Tyzzerella3* (*genus*) significantly increased the risk of hydrocephalus (OR, 36.41; 95%CI, 5.42–244.42; *P* = 2.15E-04; *P*_FDR_ = 2.37E-03), with *Candidatus Soleaferrea* (*genus*) and *Oxalobacteraceae* (*genus*) playing potential promoting roles (**Figure 6** and **Supplementary Table S15**).

When analyzing GM as an overall exposure, we found no causal relationship with any type of hydrocephalus, and sensitivity analyses revealed no significant outliers. Due to the limited number of SNPs, we could not perform further tests of heterogeneity and horizontal pleiotropy when analyzing GM separately as an exposure (**Supplementary Tables S16** and **S17**). Therefore, these causal relationships could not be confirmed under more stringent SNP screening thresholds.

### 3.4 Meta-analyses for causal relationship of hydrocephalus

To reconcile potential discrepancies when GM was the exposure and hydrocephalus from different sources (FinnGen and UK Biobank) was the outcome, we used meta-analysis. As shown in **Supplementary Table 18**, When the threshold for targeting SNPs was *P*< 1 × 10^-5^, *Melainabacteria* (*class*) (OR, 1.32; 95%CI, 1.04–1.70; *P* = 2.38E-02), *Eubacterium ruminantium group* (*genus*) (OR, 1.31; 95%CI, 1.08–1.59; P = 6.30E-03), *Haemophilus* (*genus*) (OR, 1.14; 95%CI, 1.07–1.85; *P* =1.31E-02) and *Lachnospiraceae UCG004* (*genus*) (OR, 1.66; 95%CI, 1.14–2.43; *P* = 8.10E-03) still retained the causal relationships and all served as potential risk factors for hydrocephalus. However, when the threshold was *P*< 5 × 10^-8^, *Oxalobacteraceae* (*family*) and *Tyzzerella3* (*genus*) no longer hold causal relationships.

### 3.5 Causal relationship between inflammatory factors and hydrocephalus

**Figure 7:**
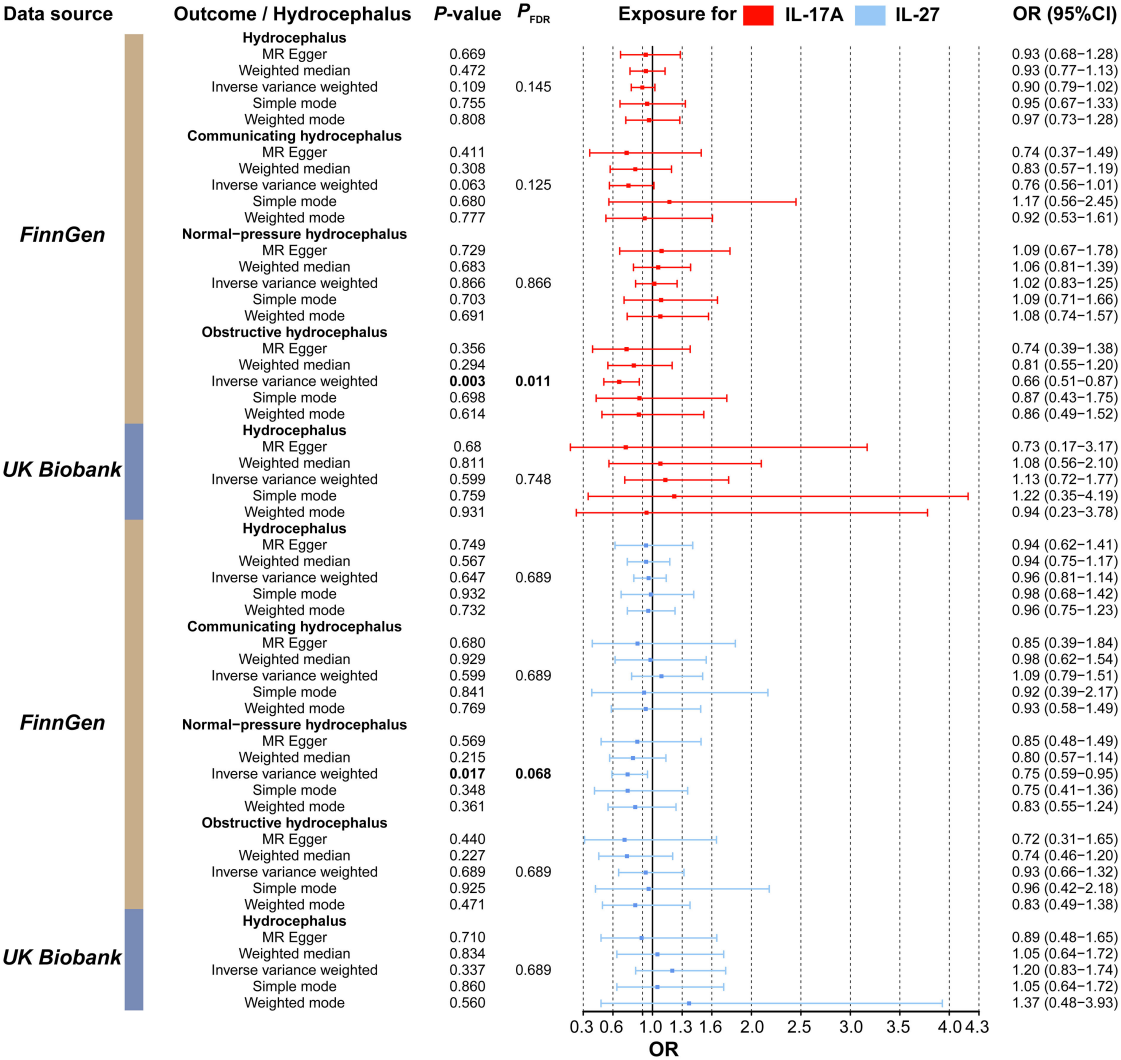
Forest plot of the association between the inflammatory factors (IL-17A and IL-27) and types of hydrocephalus. Red represents MR results when IL-17A was used as the exposure, while blue represents results when IL-27 was used as the exposure. MR, Mendelian randomization; CI, Confidence interval; OR, Odds ratio.

We then evaluated the association between inflammatory factors and hydrocephalus using two-sample MR analysis. As shown in **Supplementary Table S19**, we used IL-17A and IL-27 as exposures and identified IVs that met the criteria. After removing confounder-associated SNPs (rs10774624) (**Supplementary Table S20**), our results indicated that IL-17A (OR, 0.66; 95%CI, 0.51–0.87; *P* = 2.63E-03; *P*_FDR_ = 1.32E-02) and IL-27 (OR, 0.75; 95%CI, 0.59–0.95; *P* = 1.70E-02; *P*_FDR_ = 8.50E-02) were significantly associated with lower risks of obstructive hydrocephalus and normal-pressure hydrocephalus, respectively (**Figure 7** and **Supplementary Table S21**). In the MR results indicating causality, sensitivity analyses did not reveal significant heterogeneity, pleiotropy, or abnormal individual IVs (**Supplementary Tables S22** and **S23**).

### 3.6 Causal relationship between selected gut microbiota and inflammatory factors

**Figure 8:**
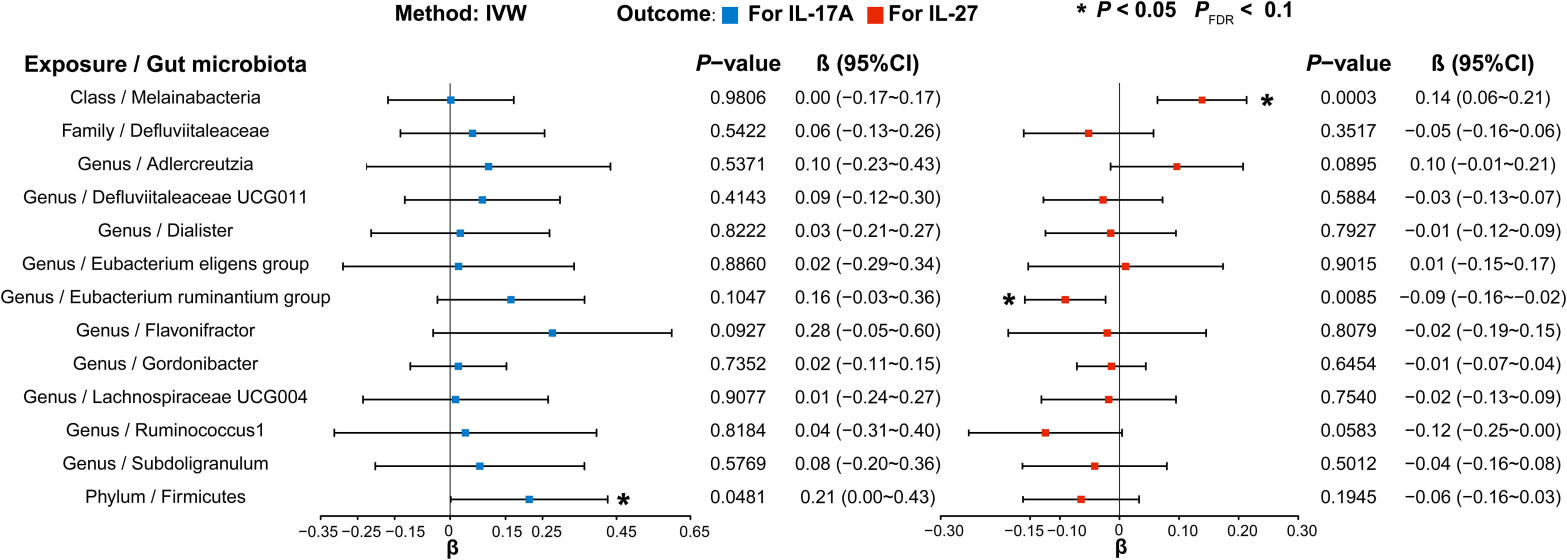
Forest plot illustrating the causal effects of gut microbiota on inflammatory factors using IVW methods. Blue (left) represents MR analysis results when IL-17A served as the outcome, and red (right) represents the results when IL-27 acted as the outcome. IVW, Inverse variance weighted.

Next, we selected GMs causally associated with normal-pressure hydrocephalus and obstructive hydrocephalus, given that IL-17A and IL-27 are known to be associated with these conditions. In our evaluation of IL-17A, we found that a significantly increased abundance of *Firmicutes* (*phylum*) (β, 0.214; 95%CI, 0.002 ~ 0.426; *P* = 4.81E-02; *P*_FDR_ = 4.81E-02) led to elevated IL-17A levels. Concerning IL-27, we observed that *Melainabacteria* (*class*) (β, 0.139; 95%CI, 0.064 ~ 0.213; *P*= 2.83E-04; *P*_FDR_ = 2.83E-04) significantly increased IL-27 levels. Conversely, the *Eubacterium ruminantium group* (*genus*) (β, -0.091; 95%CI, -0.159~-0.023; *P* = 8.47E-03; *P*_FDR_ = 8.47E-02) had the inverse effect, resulting in significantly decreased IL-27 levels (**Figure 8**, **Supplementary Tables S24** and **S25**).

In assessments of causal relationships, our sensitivity analysis revealed no significant heterogeneity and evidence of horizontal pleiotropy. Additionally, the leave-one-out analysis confirmed that no single instrumental variable significantly impacted the identified causal associations (**Supplementary Tables S26** and **S27**).

### 3.7 Causal relationship between exposures and hydrocephalus using MVMR

To further evaluate the independent effects of inflammatory factors on hydrocephalus, we utilized MVMR analysis. After removing SNP (rs10774624) associated with confounders, results indicated that IL-17A (OR, 0.35; 95%CI, 0.18–0.68; *P* = 1.97E-03) still significantly reduced the risk of obstructive hydrocephalus, and IL-27 (OR, 0.75; 95%CI, 0.58–0.97; *P* = 2.96E-02) also remained a significantly protective factor against normal-pressure hydrocephalus (shown in **Table 1**). The absence of heterogeneity and horizontal pleiotropy further strengthened the reliability of the results (**Supplementary Table S28**).

**Table 1:** Multivariable mendelian randomization analyses results between gut microbiota, inflammatory factors, and hydrocephalus.

Type of hydrocephalus/outcome	Exposure	nSNP	Methods of multivariable MR	Beta	SE	*P*-value	OR (95%CI)
Obstructive hydrocephalus	IL-17A	31	Multivariable IVW	-1.046	0.338	**1.97E-03**	0.35 (0.18–0.68)
Obstructive hydrocephalus	Phylum/Firmicutes		Multivariable IVW	-0.396	0.174	2.29E-02	0.67 (0.48–0.95)
Obstructive hydrocephalus	IL-17A		Multivariable Median	-1.160	0.495	1.92E-02	0.31 (0.12–0.83)
Obstructive hydrocephalus	Phylum/Firmicutes		Multivariable Median	-0.233	0.257	3.66E-01	0.79 (0.48–1.31)
Obstructive hydrocephalus	IL-17A		Multivariable Egger	-1.399	0.506	5.66E-03	0.25 (0.09–0.66)
Obstructive hydrocephalus	Phylum/Firmicutes		Multivariable Egger	-0.461	0.188	1.40E-02	0.63 (0.44–0.91)
Normal-pressure hydrocephalus	IL-27	40	Multivariable IVW	-0.291	0.134	**2.96E-02**	0.75 (0.58–0.97)
Normal-pressure hydrocephalus	Genus/Eubacterium ruminantium group		Multivariable IVW	0.281	0.163	8.46E-02	1.32 (0.96–1.82)
Normal-pressure hydrocephalus	IL-27		Multivariable Median	-0.261	0.186	1.60E-01	0.77 (0.54–1.11)
Normal-pressure hydrocephalus	Genus/Eubacterium ruminantium group		Multivariable Median	0.164	0.239	4.92E-01	1.18 (0.74–1.88)
Normal-pressure hydrocephalus	IL-27		Multivariable Egger	-0.363	0.216	9.33E-02	0.70 (0.46–1.06)
Normal-pressure hydrocephalus	Genus/Eubacterium ruminantium group		Multivariable Egger	0.296	0.168	7.89E-02	1.34 (0.97–1.87)

MR, Mendelian randomization; SNP, single-nucleotide polymorphism; SE, standard error; OR, odds ratio. IVW; Inverse variance weighted.

Multivariate MR analysis after removing confounder-related SNP (rs10774624).

*P*-values with significant causality are in bold.

### 3.8 Mediated MR analysis

When IL-27 was employed as a mediator, we observed a causal relationship between *Melainabacteria* (*class*) and IL-27. However, this relationship exhibited a different direction compared to the β_Total_ (*Melainabacteria/class* and hydrocephalus). Consequently, IL-27 could not mediate the causal association between *Melainabacteria* (*class*) and hydrocephalus.

**Table 2** and **Figure 2B** displayed that IL-17A played a noteworthy role in the causal pathway from *Firmicutes* (*phylum*) to obstructive hydrocephalus (with 21.01% mediated effect). While there was only a potential causal relationship between *Eubacterium ruminantium group* (*genus*) and normal-pressure hydrocephalus, L-27 may also play an essential role in the causal pathway from *Eubacterium ruminantium group* (*genus*) to normal-pressure hydrocephalus (with 7.48% mediated effect).

**Table 2:** The results of Mediation Mendelian randomization.

Exposure	Mediation	Outcome	β_Total_	β_XZ_	β_ZY_	Mediating effect	Percentage of the mediating effect
Phylum/Firmicutes	IL-17A	Obstructive hydrocephalus	-1.065	0.214	-1.046	-0.224	21.01%
Genus/Eubacterium ruminantium group	IL-27	Normal-pressure hydrocephalus	0.354	-0.091	-0.291	0.026	7.48%

β_Total_, the effect of exposure on the outcome; β_XZ_, the effect of exposure on mediation; β_ZY_, the effect of mediation on the outcome of multivariable MR analyses.

## 4 Discussion

To the best of our knowledge, this is the initial study exploring causal relationships among human GM, inflammation, and hydrocephalus. A series of MR analyses enabled us to definitively identify 4 different GMs as potentially causally associated with hydrocephalus. When focusing on the classifications of hydrocephalus, we identified 17 different GMs that had potential causal relationships with different types of hydrocephalus. In addition, *Firmicutes* (*phylum*) demonstrated a significant protective effect against obstructive hydrocephalus. We did not observe a causal relationship between hydrocephalus and the GM. Furthermore, we found that IL-17A and IL-27 reduced the risk of obstructive hydrocephalus and normal-pressure hydrocephalus, respectively. Finally, mediation analyses provided insight into the fact that *Firmicutes* (*phylum*) reduced the risk of obstructive hydrocephalus by increasing the concentrations of IL-17A, whereas *Eubacterium ruminantium group* (*genus*) potentially increased the risk of normal-pressure hydrocephalus by decreasing the concentrations of IL-27. In conclusion, we have linked GM, inflammatory factors, and hydrocephalus, which provides a foundation for exploring the mechanisms of hydrocephalus and its prevention and treatment.

GM can connect to the brain in multiple mechanisms, including metabolites, extracellular vehicles (EVs), and inflammation. First, GM can produce various tryptophan metabolites, including indole compounds. Indoles can affect brain function and behavior when they enter the circulation. Administration of indole to the cecum of rats increased blinking frequency, c-Fos expression in the dorsal vagal complex, and anxiety behavior (32). The abundance of GM-producing short-chain fatty acids (SCFAs) was reduced in rat models of bilateral carotid artery occlusion. Behavioral abnormalities and hippocampal neuronal apoptosis in rats were significantly ameliorated by transplantation of the GM-producing SCFAs (33). In patients with bipolar disorder, various metabolites, including kynurenic acid, gamma-aminobutyric acid, and SCFAs, differed significantly from controls. These metabolites were influenced by the abundance of GM, which included *Akkermansia muciniphila*, *Citrobacter freundii*, *Enterobacter cloacae*, and *Enterobacter kobei* (34). Second, EVs have been implicated as potent markers in various disease processes (35, 36). GM-derived EVs act as novel communication modes that increase tau phosphorylation and lead to cognitive impairment (37). EVs from *Pseudomonas aeruginosa* contain lipopolysaccharides (LPS), which can induce a potent neuroinflammatory response (38). In addition, GM may also regulate hippocampal and axonal development by affecting miRNA expression (an important EV component) in the hippocampal region through kynurenine pathway enzyme (39, 40).

Inflammation also acts as a modulator of disease states, extensively linking GM and the nervous system. The abundance of *Lactococcus* and *Ligilactobacillus* was found to be strongly correlated with a high-fat diet. Following an inhibitory high-fat diet, a decrease in their abundance inhibited astrocyte activity in mouse neural tissue, attenuating neuroinflammation (41). Administering ampicillin increases the abundance of *Klebsiella oxytoca* in the mouse intestine, leading to the aggregation of microglia, monocytes, and dendritic cells in the hippocampus. At this time, nuclear factor kappa-B, IL-1β, and tumor necrosis factor (TNF)-α in the brain will be significantly increased, promoting the anxiety state of mice, and administering *L. reuter*i can significantly improve this condition (42). LPS acts as a receptor involved in various inflammatory responses *in vivo*. In Parkinson’s patients, the abundance of butyric acid-producing and anti-inflammatory genera (*Blautia*, *Coprococcus*, and *Roseburia*) is significantly lower, whereas the abundance of LPS-producing Oscillospira and Bacteroides is higher. Additionally, *Bacteroides* cell wall components contain LPS, which activates the immune system via Toll-like receptor 4 (TLR4), triggering autoimmune responses (43). These findings illustrate the complex relationship between GM, inflammation, and neurological disease (44). However, previous basic and clinical retrospective studies were limited by translation issues and the direction of causation, preventing an accurate link between GM and inflammation in hydrocephalus patients. Using Mendelian randomization (MR), this complex association can be initially explored. We found a link between GM, inflammation, and hydrocephalus. Specifically, the *Firmicutes* (*phylum*) could reduce the risk of obstructive hydrocephalus by increasing the levels of IL-17A, while the *Eubacterium ruminantium group* (*genus*) potentially increased the risk of normal-pressure hydrocephalus by decreasing the levels of IL-27.

Recent studies suggest that inflammatory activation plays a crucial role in the development of hydrocephalus. Firstly, in hemorrhage-induced hydrocephalus, large amounts of hemoglobin, its degradation products, and lipopolysaccharides act as inflammatory activators. This leads to the polarization of microglial cells (M2 type), activation of resident immune cells and infiltrating macrophages, promoting the release of cytokines and chemokines, leukocyte recruitment, increased vascular permeability, and cerebral edema formation, with potential direct neurotoxic effects (45). Additionally, the choroid plexus consists of an epithelial cell membrane that includes both immune and mesenchymal cells. As a site of CSF production, the choroid plexus is critical for collecting CSF and blood (46). At the choroid plexus-CSF interface, activated cytokines exacerbate hydrocephalus by activating the phosphorylation of TNF receptor-associated stress-activated protein kinases, which leads to increased CSF production by choroid epithelial cells (3). The expression of aquaporin is upregulated during inflammatory cascade activation, further exacerbating ventricular burden (47). Elevated levels of inflammatory markers chitinase-3-like protein 1 and soluble triggering receptor expressed on myeloid cells 2 have been found in the CSF of patients with idiopathic normal-pressure hydrocephalus (48). On the other hand, adenosine, known for its neuroprotective properties, significantly decreased in the peripheral blood of affected individuals (49). These findings provide further evidence for the involvement of inflammatory components in the development and progression of hydrocephalus.

To explore the pathophysiological mechanisms of inflammatory factors in hydrocephalus, we analyzed IL-17A and IL-27, key players in inflammatory activation and immunoprotection. Rigorous MR analysis revealed that IL-17A reduces the risk of obstructive hydrocephalus, while IL-27 reduces the risk of normal-pressure hydrocephalus. IL-17A is primarily derived from TH17 cells, with various factors affecting its release. A hallmark function of IL-17A is the induction of cytokine (50). IL-17A induces inflammatory factors such as C-X-C motif chemokine ligands 1 and 2, granulocyte colony-stimulating factor, and IL-6 in acute or chronic infections. When its regulatory capacity is exceeded, IL-17A leads to pathogenic inflammation (51). IL-17A plays a well-defined role in the pathology of psoriasis, psoriatic arthritis, and ankylosing spondylitis. Dysregulated IL-17A responses are considered a risk factor for these diseases (52, 53). However, IL-17A also induces the release of antimicrobial peptides such as β-defensins and S100A8. IL-17A protects the host from microbial infections and increases the barrier function of local epithelia, such as the intestinal epithelium (54). Colonization of commensal bacteria, including Staphylococcus epidermidis, induces non-classical MHC Ib-activated Tc17 cells to produce IL-17A locally in the skin without causing significant inflammation (55). IL-17A can also induce the production of the Hypoxia-Inducible Factor 1-alpha, a re-epithelialization factor controlled by RORγt + cells, through the extracellular signal-regulated kinase (ERK) 1/2-protein kinase b-mammalian target of rapamycin pathway (8). Thus, IL-17A plays an essential role in epithelial repair after wounding. In addition, IL-17A utilizes the ERK1/2 pathway to upregulate the expression of vascular endothelial growth factor, promoting the proliferation of lymphatic vessel endothelial cells and facilitating lymphatic vessel growth (56, 57). Numerous studies have demonstrated the importance of lymphatic drainage in the resorption of hydrocephalus (58). Therefore, enhancing infection barrier function and promoting lymphatic drainage may explain the protective effect of IL-17A on obstructive hydrocephalus. IL-27 is a pleiotropic cytokine released primarily by antigen-presenting cells, capable of exerting both anti-inflammatory and pro-inflammatory effects. It has been demonstrated that IL-27 not only antagonizes IL-2 production by T cells (59), but also assists IL-10 production by Th1, Th2, and Th17 cells, thereby limiting the response of helper cells during neuroinflammation (60). When administered directly to the brain in an inflammatory state, IL-27 may inhibit granulocyte-macrophage colony-stimulating factor in CD4+ T cells and induce PD-L1 mRNA in central inflammatory cells, ultimately inhibiting autoimmune encephalomyelitis development (61). Furthermore, An IL-27-producing innate B-1a cell (i27-Breg), which accumulates and is maintained in the CNS and lymphoid tissues during neuroinflammation, exerts a protective effect against CNS autoimmune diseases. Specifically, i27-Breg immunotherapy upregulates inhibitory receptors (Lag3, PD-1) and converts conventional B cells in the CNS into lymphocytes that secrete neuroprotective IL-10 and IL-35 (62). A range of inflammatory protective effects explains the reduced risk of normal-pressure hydrocephalus associated with IL-27.

We first confirmed that *Firmicutes* (*phylum*) reduces the risk of obstructive hydrocephalus by increasing the levels of IL-17A. Specifically, *segmented filamentous bacilli* (SFB) are widely distributed Gram-positive clostridia within the *Firmicutes* (*phylum*). Studies have shown that SFB can promote dendritic cell migration in mesenteric lymph nodes via its antigen, activating RORγt+ T cells and promoting IL-17A secretion (63). As mentioned earlier, IL-17A promotes barrier function to protect the host from harmful microorganisms and acts on intestinal epithelial cells to trigger antimicrobial peptide release, maintaining intestinal homeostasis (64). Meanwhile, IL-17A released by *Firmicutes* promotes the production of lymphatic endothelium. Additionally, *Clostridium butyricum* (a member of *Firmicutes*) activates the TLR-2/ERK-AP-1 kinase pathway and promotes the release of transforming growth factor-β (TGF-β) (65). whereas upregulation of TGF-β prevents neuroinflammatory infiltration and demyelination (66). Thus, *Firmicutes* (*phylum*) is inextricably linked to inflammation and may reduce the risk of obstructive hydrocephalus by enhancing the gut barrier and lymph angiogenesis via IL-17A. We also found that the *Eubacterium ruminantium group* (*genus*) potentially increases the risk of normal-pressure hydrocephalus by decreasing the levels of IL-27. *Eubacterium groups* play a broad regulatory role in host inflammation. *Eubacterium* is reported to be the main SCFA-producing group. SCFA promotes the activation of G protein-coupled receptor 109A, further activating macrophages and dendritic cells, which express free fatty acid receptor 2 and block IL-27 expression (67, 68). The relationship between *Eubacterium ruminantium group* (*genus*) and IL-27 remains unclear. Recent studies have found that *Eubacterium ruminantium* can reduce the expression of inflammatory factors, such as IL-1β and TNF-α (69). However, the *Eubacterium ruminantium group* (*genus*) also upregulates extracellular matrix protein 1 in bladder tissues, increasing bladder cancer risk by raising matrix metalloproteinase 9 expression through the ERK1/2 phosphorylation pathway (70). Thus, *Eubacterium ruminantium group* (*genus*) may act as a pleiotropic bacterium with a complex association with inflammatory factors. Its inhibition of certain inflammatory factors may also inhibit IL-27, which further may lead to the dysregulation of inflammatory factors, thereby promoting the development of normal-pressure hydrocephalus.

Our analyses still have some limitations. First, all data sources were from European populations, which limits the generalization of the results to other ethnic populations. Therefore, more refined cohort studies of different ancestries are needed to increase the generalizability of the results. Second, the MR analyses used summarized data, which did not allow us to perform nonlinear correlation analyses. Third, since the selection of SNPs with *P<* 5 × 10^-8^ was too limited for the GM data, we chose SNPs with *P*< 1 × 10^-5^ as the IVs of GM for analysis. Subsequently, we screened a series of SNPs to minimize the spurious associations introduced by the loose threshold. Finally, the minimal categorization of the dataset for GM limited our more microscopic assessment of causality. Nonetheless, we established a relationship between GM, inflammatory factors, and various types of hydrocephalus through multiple MR analyses, laying the foundation for further research.

## 5 Conclusion

We identified 4 different taxa of GM with potential causal effects on hydrocephalus and 16 taxa of GM as potentially causal for different classifications of hydrocephalus. Furthermore, *Firmicutes* (*phylum*) reduced the risk of obstructive hydrocephalus by increasing the concentrations of IL-17A, whereas the *Eubacterium ruminantium group* (*genus*) potentially increased the risk of normal-pressure hydrocephalus by decreasing the concentrations of IL-27. Therefore, modifying the abundance of GM could potentially alter the inflammatory state of the organism, thereby reducing the risk of hydrocephalus. Our study lays an essential foundation for explaining the mechanisms of the gut-brain axis. However, more refined basic experiments and clinical trials are still necessary.

## Data availability statement

The original contributions presented in the study are included in the article/**Supplementary Material**. Further inquiries can be directed to the corresponding authors.

## Author contributions

YS: Conceptualization, Data curation, Formal analysis, Methodology, Software, Visualization, Writing – original draft. CL: Conceptualization, Software, Writing – review & editing. XZ: Data curation, Investigation, Visualization, Writing – review & editing. YW: Resources, Software, Visualization, Writing – review & editing. HZ: Conceptualization, Resources, Validation, Writing – review & editing. ZY: Software, Writing – review & editing. BG: Formal analysis, Validation, Writing – review & editing. RH: Investigation, Resources, Writing – review & editing. QL: Software, Visualization, Writing – review & editing. AG: Project administration, Supervision, Writing – review & editing. HL: Funding acquisition, Project administration, Supervision, Writing – review & editing.
